# Fabrication and optimization of Nanodiamonds-composited poly(ε-caprolactone) fibrous matrices for potential regeneration of hard tissues

**DOI:** 10.1186/s40824-018-0126-x

**Published:** 2018-05-30

**Authors:** Guk Young Ahn, Tae-Kyung Ryu, Yu Ri Choi, Ju Ri Park, Min Jeong Lee, Sung-Wook Choi

**Affiliations:** Department of Biotechnology, The Catholic University of Korea, 43 Jibong-ro, Wonmi-gu, Bucheon-si, Gyeonggi-do 420-743 Republic of Korea

**Keywords:** Biodegradable polymer, Composite, Nanodiamond, Electrospinning, Guided tissue engineering

## Abstract

**Background:**

Electrospun fibrous matrices are of great importance for tissue engineering and drug delivery device. However, relatively low mechanical strength of the fibrous matrix is one of the major disadvantages. NDs with a positive charge were selected to enhance the mechanical property of a composited fibrous matrix by inducing the intermolecular interaction between NDs and polymer chain. We prepared ND-composited poly (ε-caprolactone) (PCL) fibrous matrices by electrospinning and evaluated their performance in terms of mechanical strength and cell behaviors.

**Methods:**

A predetermined amounts of NDs (0.5, 1, 2 and 3 wt%) were added into PCL solution in a mixture of chloroform and 2,2,2-trifluoroethanol (8:2). ND-composited PCL (ND/PCL) fibrous matrices were prepared by electrospinning method. The tensile properties of the ND/PCL fibrous matrices were analyzed by using a universal testing machine. Mouse calvaria-derived preosteoblast (MC3T3-E1) was used for cell proliferation, alkaline phosphatase (ALP) assay, and Alizarin Red S staining.

**Results:**

The diameters of the fibrous matrices were adjusted to approximately 1.8 μm by changing process variables. The intermolecular interaction between NDs and PCL polymers resulted in the increased tensile strength and the favorable interfacial adhesion in the ND/PCL fibrous matrices. The ND/PCL fibrous matrix with 1 wt% of ND had the highest tensile strength among the samples and also improved proliferation and differentiation of MC3T3-E1 cells.

**Conclusions:**

Compared to the other samples, the ND/PCL fibrous matrix with 1 wt% of ND concentration exhibited superior performances for MC3T3 cells. The ND/PCL fibrous matrix can be potentially used for bone and dental tissue engineering.

## Background

Electrospun fibrous matrices are very useful in tissue engineering because of their large surface area, high aspect ratio, porosity, and the presence of very small pore structures on the fibers [1–3]. Most importantly, the topological structure of the electrospun products can mimic the extracellular matrix and enhance both cell migration and proliferation [4]. Many researchers have demonstrated the production of fibrous structures from various organic/inorganic materials and also prepared organic/inorganic composite fibers [5, 6]. Furthermore, several groups have investigated composite fibers with a therapeutic agent and functional materials using the electrospinning method. Zhang et al. fabricated electrospun biomimetic composited chitosan nanofibers containing hydroxyapatite (HAp) and confirmed the bone forming ability as shown by the cell proliferation, mineral deposition and morphology observation [7]. Li et al. designed and evaluated the Silk fibroin fibrous scaffolds containing bone morphogenetic protein-2 and HAp nanopowders for bone tissue engineering [8]. Ma et al. reported enzymatic degradable hydrogels based on collagen and alendronate conjugated HAp nanoparticles. These hydrogels demonstrated excellent biocompatibility and promoted the adhesion and proliferation of MC3T3-E1 cells [9]. Yang et al. were successful in coating electrospun poly(ε-caprolactone) (PCL) with a thin layer of calcium phosphate for bone tissue engineering [10]. Rajzer et al. prepared an electrospun bi-layer fibrous scaffold using PCL and gelatin modified with calcium phosphate for bone mineralization [11]. Cao et al. fabricated beta-tricalcium phosphate (β-TCP) composited poly(glycolic acid) three-dimensional scaffolds using solvent casting and particle leaching method and evaluated their biocompatibility, osteoconductivity, osteogenesis and degradation in vivo [12]. In general, the materials for the enhancement of osteoconduction have been limited to inorganics such as HAp and calcium phosphates.

Carbon-based materials, including fullerene, graphene, carbon nanotubes, graphite, nanohorns and nanodiamonds (NDs), have also been investigated for various biomedical applications [13, 14]. Depan et al. investigated the biological response of graphene conjugated chitosan scaffolds and reported their higher mechanical properties, lower degradation rate, and enhanced osteoblast cell growth [15]. Pan et al. prepared the multiwall carbon nanotubes/PCL composite scaffolds using solution evaporation technique and evaluated the proliferation and differentiation of bone marrow stromal cells [16]. Recently, NDs, which are carbon-based allotrope nanoparticles of truncated octahedral composition, have attracted attention as an innovative nanomaterial because of their high biocompatibility, spherical morphology, high density, surface functionality, and strong hardness [17]. Many researchers have reported on the excellent non-toxicity and biocompatibility of NDs using a variety of cells such as epithelial cell, adenocarcinoma cell, neuroblastoma cell, and so on [18, 19]. Grausova et al. designed silicon films with NDs and confirmed the enhanced adhesion, spreading, viability, growth, and maturation of human osteoblast-like MG63 cells on these films [20]. Zhang et al. fabricated fluorescent poly(L-lactic acid)-ND composite thin film and demonstrated the enhanced proliferation and differentiation of osteoblasts on this film as well as an increased mechanical strength of the scaffold [21]. In view of these interesting applications, a systematic study of the effect of NDs used as composite materials for tissue engineering is crucial.

Recently, our group prepared bone-targeted drug carriers conjugated with NDs and found that NDs itself increased the alkaline phosphatase (ALP) activity [22]. By inspiring our previous results, NDs with a positive charge were composited in a fibrous matrix in an effort to enhance the mechanical strength and cellular behaviors on the fibrous matrix. We fabricated ND-composited PCL (ND/PCL) matrices using the electrospinning method for guided tissue engineering and optimized the ND concentrations in terms of the tensile properties of the matrix and the proliferation and differentiation of preosteoblast. We believe that the PCL/ND matrices have great potential applications for regeneration of hard tissues.

## Methods

### Preparation of ND/PCL fibrous matrices

Fibrous matrices were produced using a horizontal electrospinning setup [23]. Polycaprolactone (PCL, *M*_w_ = 80,000, Sigma–Aldrich), chloroform (CF, Sigma–Aldrich), and 2,2,2-trifluoroethanol (TFE, Sigma-Aldrich) were used to fabricate the ND/PCL fibrous matrices. NDs with a positive charge (46.0 ± 3.4 mV) were purchased from Neomond Ltd. (Bucheon, Korea). The NDs were analyzed using the Zetasizer (Malvern Instruments Ltd., Worcestershire, UK) to determine their zeta potentials. For the preparation of the PCL fibrous matrix, PCL solution (10 mL, 10 wt%, dissolved in CF:TFE = 8:2) was electrospun onto the aluminum dish using a syringe pump (NE-1000, New Era Pump Systems Inc., New York, USA). A high voltage was applied between the spinneret (a 24 G needle) and the collector located 15 cm away [24]. The syringe was horizontally fixed on the syringe pump and the PCL and ND/PCL solutions were electrospun onto an aluminum foil collector. The ND concentration of the ND/PCL solution was varied as 0.5, 1, 2, and 3 wt%. In order to ensure that their diameter were similar, the fibers were fabricated under different conditions of applied voltage and flow rate [25].

### Characterization of the ND/PCL fibrous matrix

Scanning electron microscopy (SEM, S-4800, Hitachi High-Technologies, Co. Ltd., Tokyo, Japan) was used to characterize the morphologies of the ND/PCL fibrous matrices. The diameters of the fibers were calculated from the SEM images of the samples by analyzing at least 300 fibers using the ImageJ® software (National Institutes of Health, Bethesda, USA). The tensile properties of the ND/PCL fibrous matrices were analyzed by using a universal testing machine (UTM, LR 10 K, Lloyd instruments Ltd., London, UK). The dimensions of the fibrous matrices were 10 mm × 80 mm × 1 mm (width × length × thickness). The thickness of the fibrous matrix was measured with digital caliper. The extension rate was maintained at 5 mm/min at room temperature. The load cell was 50 N with a gauge length of 50 mm.

### Cell culture on the fibrous matrices

Four types of the ND/PCL fibrous matrices with ND concentrations of 0.5, 1.0, 2.0, and 3.0 wt% were prepared for the cell culture, where the PCL fibrous matrices served as a control. Each fibrous matrix was cut into square sheets of 10 mm length. Mouse calvaria-derived preosteoblast (MC3T3-E1) and mouse fibroblast (NIH/3 T3) were used for cell proliferation on the matrices. Prior to cell seeding, the five types of fibrous matrices were sterilized using 70% ethanol for 24 h, washed with PBS (Welgene) three times, and dipped in the culture medium for a day. The MC3T3-E1 and NIH/3 T3 cells with a density of 1.0 × 10^7^ cells in the media were used for cell seeding on to each fibrous matrix and cultured in Dulbecco’s Modified Eagle’s Medium (DMEM, Welgene), which contained 10% fetal bovine serum (FBS, Welgene) and 1% antibiotics (penicillin and streptomycin, Welgene). The fibrous matrices were maintained in the media for a day until the cells had adhered to them. The cell-seeded fibrous matrices were subsequently transferred to 24-well plates, and maintained in an incubator at 37 °C under a humidified atmosphere containing 5% CO_2_. The media were changed every 2 days.

Cell proliferation was measured using the Cell Counting Kit-8 (CCK-8, Dojindo, Co. Ltd.) at 1, 3, 5, and 7 days after cell seeding. The CCK-8 solution (20 μL) was added to each well of the 24-well plates containing the cell-seeded fibrous matrices and maintained in an incubator for 1.5 h [26]. The sample extracts were transferred to 96-well plates and their absorbances at 450 nm were measured by using a microplate reader (Spectramax Plus 384, Molecular Devices, Co. Ltd., Philadelphia, USA) [27].

Alkaline phosphatase (ALP) assay was performed at 1, 3, and 5 days after seeding on the fibrous matrices. The fibrous matrices were washed thrice with PBS and then immersed in 1 mL of radio-immunoprecipitation assay buffer (RIPA buffer, Thermo Scientific). Subsequently, they were stored at − 20 °C for 30 min and centrifuged for 10 min at 13000 rpm. The supernatant (50 μL) was transferred to a 96-well plate and 50 μL of *p*-nitrophenyl phosphate (pNPP, Sigma–Aldrich) solution was added to each well. The plate was kept at 37 °C for 30 min and after the addition of 50 μL of 3 N NaOH, the absorbance at 405 nm was measured using a microplate reader. The total protein content was measured and the ALP activity was calculated by dividing the *p*-nitrophenol quantitation by protein quantification. The supernatant (2 μL) was transferred to a 24-well plate and 800 μL of distilled water and 200 μL of Bio-Rad protein assay solution (Bio-Rad Laboratories) were added to each well. 100 μL of this solution was then transferred to a 96-well plate and the absorbance at 595 nm was measured using a microplate reader. Calcium deposits in the fibrous matrices were stained by Alizarin Red S (ARS, Sigma–Aldrich). The cell-seeded fibrous matrices were washed thrice with PBS, and 1 mL of 40 mM ARS was added to each well and kept at 37 °C for 30 min. Subsequently, the fibrous matrices were washed with distilled water five times and observed by an inverted microscope (IX71-F22PH, Olympus, Tokyo, Japan) [28].

### Statistics

All experimental data were expressed as means ± standard deviation (s.d.). Statistical analysis was evaluated by analysis of variance (ANOVA). The statistical significance was set at *p* < 0.05.

## Results

**Fig. 1:**
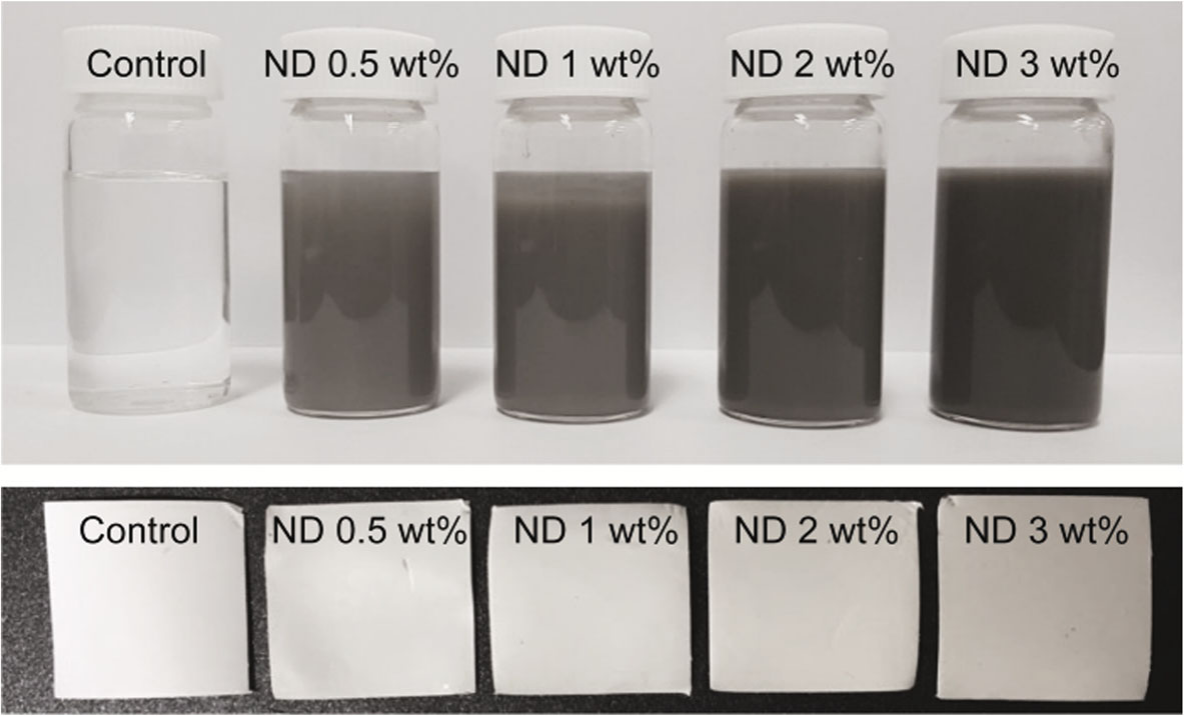
Photographs of the PCL solutions and the corresponding ND/PCL matrices with varying ND concentration (0.5, 1, 2, and 3 wt%). The PCL solution and fibrous matrices were used as a control

**Fig. 2:**
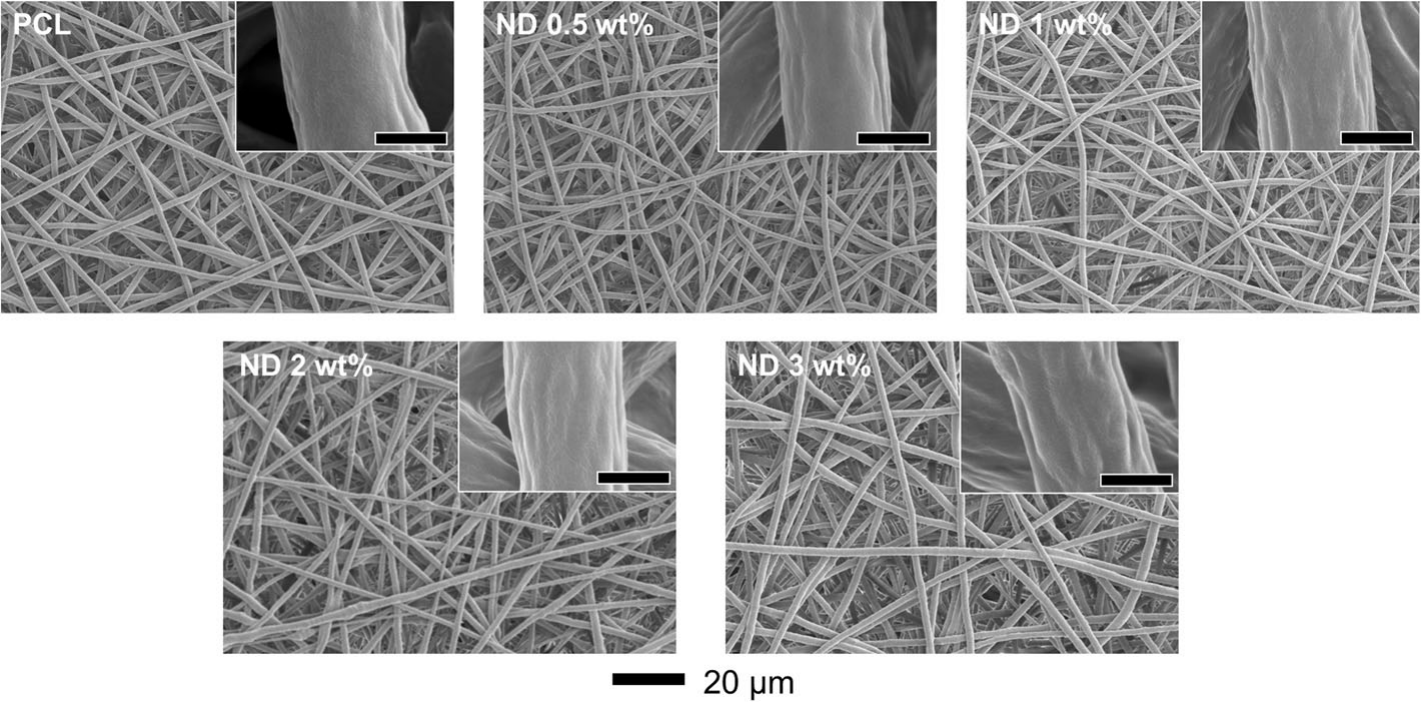
SEM images of the PCL and ND/PCL fibrous matrices with different ND concentrations. The insets are magnified SEM images and the scale bars are 1 μm

To prepare ND/PCL fibrous matrices, a predetermined amount of ND powders (0.5, 1, 2, and 3 wt% relative to the amount of PCL) was added into the PCL organic solution (10 wt%), followed by ultrasonication in an ice bath for a homogeneous dispersion. As shown in Fig. 1, the ND/PCL dispersion and electrospun fibrous matrices exhibited a darker gray color with the increasing ND concentration, which was because of the intrinsic color of NDs. The higher contrast of the ND/PCL fibrous matrices with the higher ND concentrations suggested the successful incorporation of NDs in each matrix. Figure 2 shows the SEM images of the resultant PCL and ND/PCL matrices with different ND concentrations. The fibers of the PCL and ND/PCL matrices were randomly deposited. Fiber diameter is one of the key factors that affects the properties of fibrous matrices and cell activities [29, 30]. In this work, to evaluate the effect of ND concentration on the tensile strength and cellular activity, the diameters of PCL and ND/PCL fibers were adjusted to approximately 1.8 μm by changing the flow rate and applied voltage in the electrospinning setup, because relatively thick fiber can facilitate cell proliferation [31, 32]. The detailed synthetic conditions and average diameter of the fibers are presented in Table 1.

**Table 1:** Fabrication conditions of the PCL and ND/PCL fibrous matrices with varying ND concentration (0, 0.5, 1, 2, and 3 wt%). The fiber diameter of the PCL and ND/PCL fibrous matrices was determined using ImageJ® software

Groups	Fiber diameter (μm)	Voltage (kV)	Flow rate (mL/min)
PCL only	1.82 ± 0.12	10	0.008
ND 0.5 wt%	1.93 ± 0.13	8.5	0.02
ND 1 wt%	1.83 ± 0.16	8	0.03
ND 2 wt%	1.84 ± 0.25	7.5	0.03
ND 3 wt%	1.80 ± 0.24	6.5	0.03

**Fig. 3:**
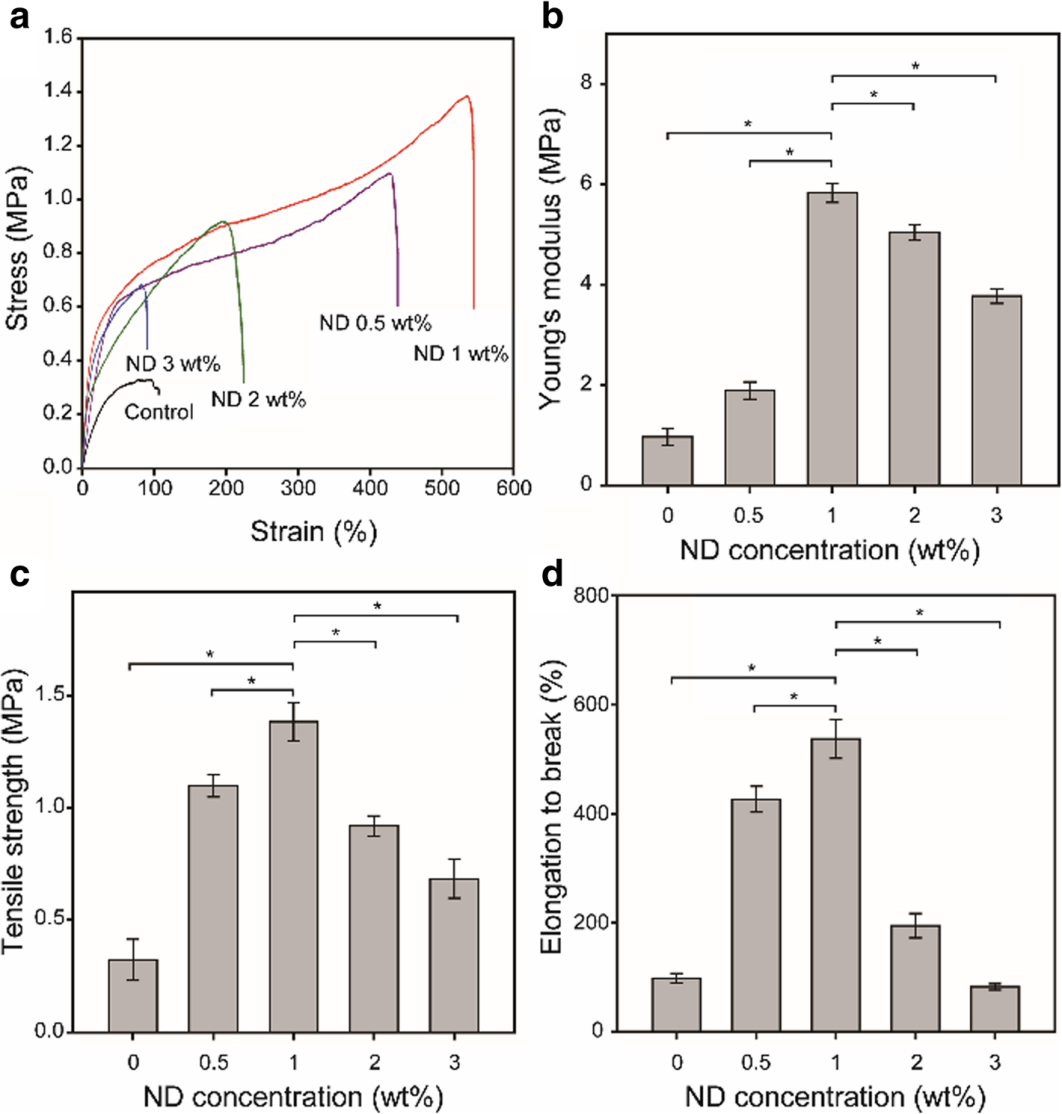
**a** Representative strain–stress curves of the ND/PCL fibrous matrices with different ND concentration (0, 0.5, 1, 2, and 3 wt%). **b** Young’s modulus, **c** tensile strength, and **d** elongation at break of the PCL and ND/PCL fibrous matrices. * Significant difference between the two groups (*p* < 0.05)

Figure 3a shows the representative strain-stress curves of the PCL and ND/PCL fibrous matrices. The ND/PCL fibrous matrices with ND concentrations less than 2 wt% showed more elongation as compared to the PCL fibrous matrix. Note that the ND/PCL fibrous matrix with 1 wt% of ND exhibited the most elongated and strongest tensile properties. All the tensile properties, including Young’s modulus (Fig. 3b), tensile strength (Fig. 3c), and elongation at break (Fig. 3d), increased with the increasing ND concentration up to 1 wt% and declined as the ND concentration increased further. The enhanced tensile strengths were mainly attributed to the strong intermolecular interaction between NDs and PCL polymer chains [33]. During solvent evaporation in electrospinning, the aggregation of NDs in the ND/PCL fibrous matrices with a large amount of ND concentration might result in their reduced tensile properties [34, 35]. The Young’s modulus and elongation at break increased approximately six folds by compositing 1 wt% of NDs, as compared to the PCL fibrous matrix.

**Fig. 4:**
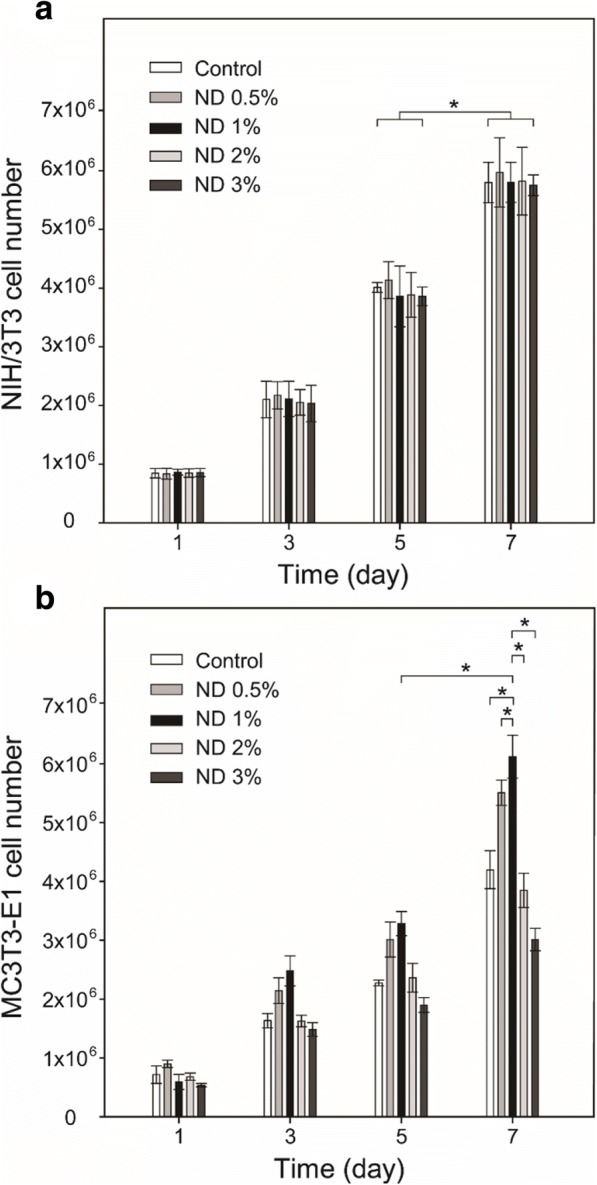
Proliferation of (**a**) NIH/3 T3 and (**b**) MC3T3-E1 cells cultured on the PCL only (control) and ND/PCL fibrous matrices with varying ND concentration (0.5, 1, 2, and 3 wt%). * Significant difference between the two groups (*p* < 0.05)

Apart from the tensile properties, in vitro cellular effects of the ND/PCL fibrous matrices were also evaluated using the NIH/3 T3 and MC3T3-E1 cells. MC3T3-E1 (mouse calvaria-derived pre-osteoblast) cell lines were chosen as the bone model cells, whereas NIH/3 T3 cells (mouse embryonic fibroblast) were used as normal model cells (control). Fig. 4 shows the cell proliferation on the PCL and ND/PCL fibrous matrices with respect to time. There was no significant difference in proliferation rate when NIH/3 T3 cells were cultured on the PCL and ND/PCL fibrous matrices. In contrast, the proliferation rate of MC3T3-E1 cells on the ND/PCL fibrous matrix with 1 wt% of ND was approximately 1.5 times that of the PCL fibrous matrix. The proliferation rates of the MC3T3-E1 cells on the ND/PCL fibrous matrices with 2 and 3 wt% of ND were lower than that on the PCL fibrous matrix, which is attributed to the strong positive surface charge of those matrices.

**Fig. 5:**
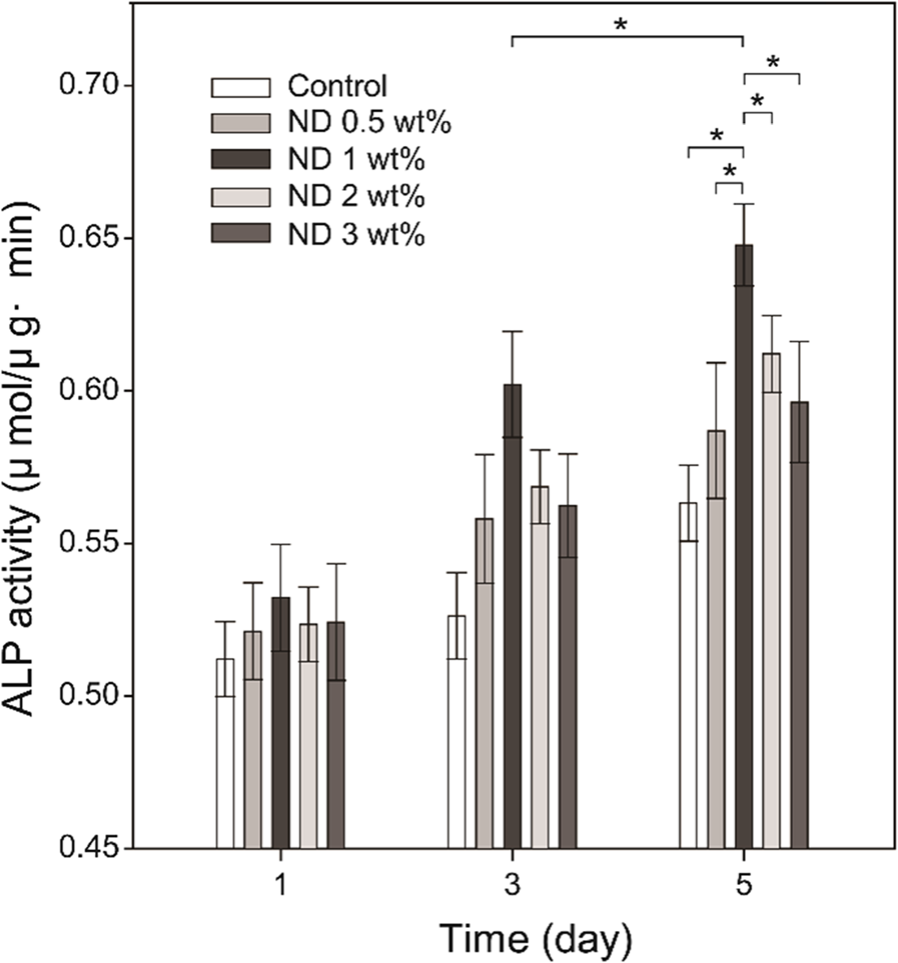
Variation of ALP activities of MC3T3-E1 cultured on the PCL and ND/PCL fibrous matrices (0.5, 1, 2, and 3 wt% ND concentration). The PCL fibrous matrices served as the control. * Significant difference between the two groups (*p* < 0.05)

**Fig. 6:**
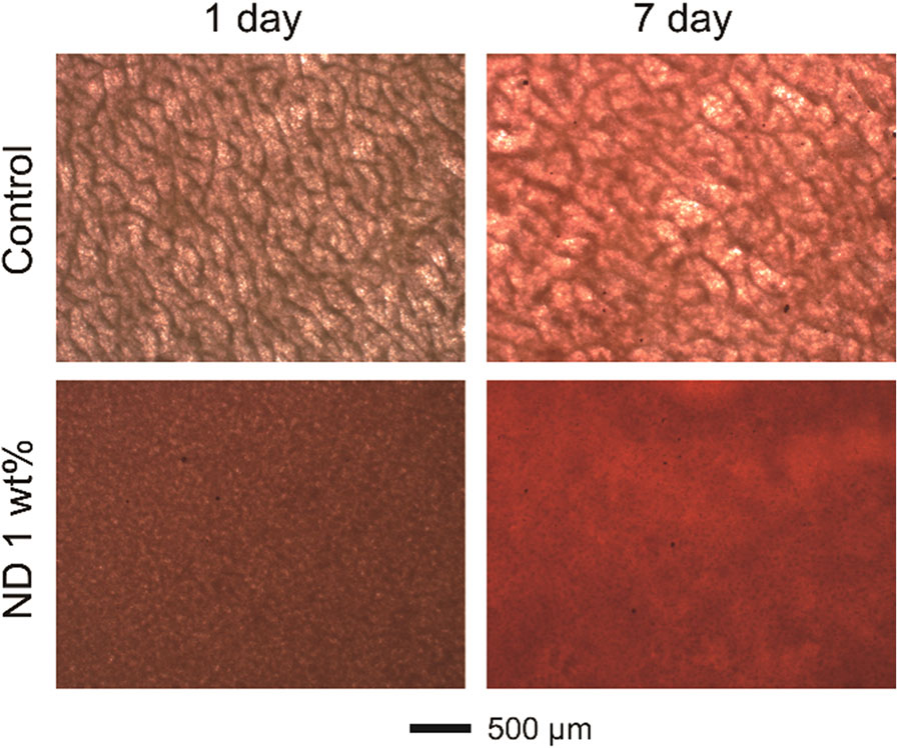
Representative optical images of the cell/fibrous matrices cultured on the PCL (control) and ND/PCL fibrous matrices (1 wt% ND concentration) for 1 and 7 days, followed by Alizarin Red S staining

To further verify the positive cellular effect of NDs in a fibrous matrix, MC3T3-E1 cells were cultured on the PCL and ND/PCL fibrous matrices and the ALP activity was measured at 1, 3, and 5 days. As shown in Fig. 5, the ND/PCL fibrous matrix increased the ALP activity as compared to the PCL fibrous matrix and the employment of 1 wt% of ND induced the highest ALP activity. By considering Figs. 4 and 5 together, it was concluded that the ND/PCL fibrous matrices with 2 and 3 wt% of ND concentrations exhibited relatively lower proliferation rates for MC3T3-E1 cells but slightly higher ALP activities in comparison with the PCL fibrous matrix, suggesting that the employment of NDs facilitated the ALP activity. In addition to the ALP activity test, calcium secreted from the differentiated MC3T3-E1 cells was stained using Alizarin Red S to visualize the calcium deposition on the fibrous matrix (Fig. 6). Red color was vividly observed on the ND/PCL fibrous matrix with 1 wt% of ND. These results confirmed that the MC3T3-E1 cells were more differentiated on the ND/PCL fibrous matrix with 1 wt% of ND and also effectively secreted minerals.

## Discussion

In this work, NDs with a high positive charge of 46.0 ± 3.4 mV were used for the ND/PCL fibrous matrices. The high positive charge could induce the intermolecular interactions (e.g., electrostatic interaction) between NDs and PCL polymer chains, leading to the favorable interfacial adhesion in composites. The NDs were well dispersed in the PCL organic solution without sedimentation for 5 h due to the nano-scale size and high zeta potential of NDs, which was enough for electrospinning. The fiber diameter of the PCL and ND/PCL fibrous matrices was controlled by the flow rate and applied voltage. Unlike the ND/PCL fibrous matrices, the relatively low flow rate for the fabrication of the PCL fibrous matrix was attributed to the electrostatic repulsive force between positive charged NDs and nozzle connected to positive voltage in electrospinning.

The PCL-based scaffolds generally had a relatively low mechanical strength as compared to other biodegradable polymers (e.g., poly(lactic acid), poly(glycolic acid), and polydioxanone), which is because of the intrinsic low glass transition temperature of PCL (approximately − 60 °C) [36]. Therefore, the mechanical properties of PCL-based scaffolds need to be enhanced in order to expand the application area. The ND/PCL fibrous matrix with 1 wt% of ND showed the best tensile properties among the samples. It is hard to directly compare the mechanical properties of other PCL-based fibrous matrix composited with inorganic materials because of the differences in the fiber diameters and the thickness of the samples. However, some comparisons can be made. The tensile strength and Young’s modulus of the PCL fibrous scaffolds with 6 wt% of nanoclay content were increased less than two-fold in comparison with the PCL-only scaffolds [37]. In addition, there was no significant difference in the tensile strength between HAp-composited PCL and PCL-only fibrous matrices, whereas the elongation at break was much increased by the employment of HAp [38]. These comparisons suggested that the employment of ND as a composited material can notably increase the mechanical strength and thus enhance the application area of PCL-based fibrous matrices. Despite the increased mechanical strength, the application of the ND/PCL fibrous matrices would be limited to guided tissue engineering of hard tissue because of their low mechanical strength as compared to other poly(lactic acid)-based scaffolds with a three-dimensional structure.

To evaluate the toxicity of NDs in the fibrous matrix, the NIH/3 T3 cells were cultured on the PCL and ND/PCL fibrous matrices. The NIH/3 T3 cells well proliferated all the fibrous matrices over time and there was no significant difference in the proliferation rate among the PCL and ND/PCL fibrous matrices, suggesting the nontoxicity of NDs even at a relatively high concentration upto 3 wt%. The higher proliferation rate on the ND/PCL fibrous matrix with 1 wt% of ND can be attributed to the fact that the bone cells favored a hard surface for proliferation [39–41]. However, the ND/PCL fibrous matrices with 2 and 3 wt% of ND concentrations exhibited slightly lower proliferation rates compared to the PCL fibrous matrix. The lower proliferation rate is attributed to the high positive surface charge of the ND/PCL fibrous matrices with relatively high ND concentrations, because the high positive surface charge generally enhanced adhesion but inhibited proliferation of cells [42]. It was implied that the cells had a more preferred range of the mechanical strength for proliferation. In addition, the ALP activity result revealed that the MC3T3-E1 cells were favorably differentiated to osteoblasts on the ND/PCL fibrous matrix with 1 wt% of ND. Taken together, the employment of 1 wt% of ND in the fibrous matrix enhanced the tensile strength of the matrix and also proliferation and ALP activity of MC3T3 cells.

## Conclusions

We fabricated NDs-composited PCL fibrous matrices using an electrospinning method. The ND/PCL fibrous matrix with 1 wt% of ND concentration demonstrated several advantages in terms of the tensile properties, proliferation and differentiation of MC3T3-E1 cells, and calcium deposition. These superior features were attributed to the provision of a favorable environment of NDs for the MC3T3-E1 cells. The ND/PCL fibrous matrix can be potentially used for guided tissue engineering of dental tissue and bone. Our next endeavors are focused on the development of a three-dimensional scaffold composited with NDs for bone replacement.
